# Health Tract, No. 54

**Published:** 1865

**Authors:** 


### HEALTH TRACT, No. 54.

### DIET FOR INVALIDS.

MANY persons, while apparently recovering from sickness, suddenly become worse and die, in consequence of eating some improper article of food, or of eating too much or too often; others have perished in eating against their inclination, merely to please their friends, or to get rid of their solicitations.

If persons are able to be out of bed, or on their feet, the intervals of eating should be about four hours during day-light. Only those confined to bed should eat oftener, or during the night. As a general rule, that is best for the patient for which there is the greatest craving. But a lady recovering from an attack of typhod fever had a strong desire to eat a sweet potato. She did so, and died next day. Hence, a very small amount of what is craved should be taken at a time; and if no discomfort follows within four hours, a little more may be ventured.

If a patient wakes up in the morning thirsty, or the mouth is dry, no solid aliment should be taken, however great the hunger. Liquid food only can be safely used, at least until near noon.

The very best restorative an invalid can swallow, when thirsty or "faint," is the very best green or black tea that money can purchase, made in the best manner; the strength to be adapted to the circumstances. If feeble, the patient should have the food as soon as possible after it is called for. If there is no appetite, remove it instantly; instead of letting it remain, in the hope of its being soon wanted. Never, under any circumstances, give one single spoonful more than the patient can take with a relish, with satisfaction. A teaspoonful every twenty minutes, taken with a will, does more good than a dozen times the amount every hour or two, when such an amount can not be taken without distaste.

BEEF-TEA. Liebig's.—Chop a pound of lean meat as fine as for sausage; mix it with a pint of cold water; put it over a slow fire; when it has boiled five minutes, strain through a coarse cloth; salt to suit.

BROTH, quickly made.—Take a bone of loin or neck of mutton; remove skin and fat; beat or cut fine the meat; cover it with water in a sauce-pan, with a cover; season it; boil quickly for half an hour.

PANADA, in five minutes.—To water and white wine, seasoned with sugar, nutmeg, and lemon-pell, grate in some bread as soon as it boils; boil fast until thick enough to drink.

SWEET BUTTERMILK.—In ten minutes after milking, churn until flakes of butter swim about thickly. Good to drink while eating crackers, rusk, ripe or dried fruits.

FLOUR CAUDLE.—Rub a tablespoonful of fine flour into six of water; add this to five spoonfuls of milk, while boiling; stir twenty minutes over a slow fire. A nourishing astringent for weak bowels.

WINE-WHEY.—While a pint of milk is boiling, stir in eight tablespoonfuls of wine; boil a minute; when curd has settled, turn off the whey, which sweeten and drink, cold or warm.

TOAST-WATER.—Toast slowly, until brown, a thin slice of the soft of stale bread; put in a pitcher; pour on boiling water, and set it to cool, covered.

WATER-GRUEL.—Make two tablespoonfuls of Indian, or corn-meal, and one of flour, into a thick batter, with cold water; stir in boiling water till suitably thick; season with salt, and stir while boiling for six or eight minutes; add a little butter, and pour it over toasted bread, cut in small pieces.

TOASTED BREAD.—Hold a thin slice to the fire until it turns slowly of a straw color on both sides.

FLAXSEED-TEA.—Boil whole flaxseed in water to a thick syrup. A dessert-spoonful to a glass of water; strain, and add sugar and lemon-juice to suit.

